# Diet

**Published:** 1883-01

**Authors:** 


### DIET.

IT is a popular idea that the majority of persons eat to much. If reference is made to the bad cooking generally in vogue in this country, it is probably true; but if it were practicable to make an estimate of the amount of food required by every individual, based on the energy expended in the mental or bodily activities of each, we believe the nutriment contained therein would rather fall below than exceed tł e reasonable requirements of the body, and that this inadequate quantity, together with its bad preparation, accounts in a great measure for much of the premature decay visible on every side. This practical age, with its gauges and tests, and its demand for reasons and for facts, has dispelled many false notions. The present generation of men cannot be made to believe that their forefathers attained an average age of eighty years by hard work and "hard tack;" but they do know that the average term of human life was ten years less in the last century than in the present.

There is no question so frequently asked of the physician as that which relates to diet; and there is none more difficult to answer. The answer almost always given, by one of the most prominent physicians in New York, is, "eat whatever you think you would relish." This reply is wise. Nature craves what will best agree with the system; and unless there is some special reason why it should be withheld, the desired food should be furnished if possible. One qualification, however, is necessary; and that is that the food requested should be wholesome and properly prepared. A patient just recovering from typhoid fever might crave an article of food to which he had always been accustomed, as for example, "mince pie,"—a dish most trying to the strongest digestive organs—and which, if then indulged in, would almost certainly result in a relapse that would prove fatal. A similar case once came under our observation as *consulting* physician. It is our firm conviction that no restrictions should ordinarily be placed on the quantity of food given, to any one, provided it is plain and nutritious food, properly cooked and taken at meal time. Young children require food more frequently, and are permanently injured by fasting between the regular meal hours. Never say no! to a child who asks food or water, it matters not how often. No person who has been habitually underfed in childhood can ever enjoy perfect health, nor is he likely to attain to old age. We see this truth exemplified in men and animals.

The use of food is not alone to nourish the body; it has another most important office, which is to distend the stomach and intestines. If a horse were to be fed on oats or other concentrated food only, he would soon die; and thus it would be with any other animal.

When a child is underfed, during years—as thousands are—its stomach becomes weak and contracted so that in time it has neither the strength nor capacity to contain sufficient nutriment for the wants of the system, and the child dies of "marasmus," a disease common enough among the poor and degraded, and not altogether unknown in higher life, where errors in relation to the care of children are sometimes adhered to with most persistent obstinacy.

